# Reviewing the Modern Therapeutical Options and the Outcomes of Sacubitril/Valsartan in Heart Failure

**DOI:** 10.3390/ijms231911336

**Published:** 2022-09-26

**Authors:** Diana-Carina Iovanovici, Simona Gabriela Bungau, Cosmin Mihai Vesa, Madalina Moisi, Elena Emilia Babes, Delia Mirela Tit, Tunde Horvath, Tapan Behl, Marius Rus

**Affiliations:** 1 Doctoral School of Biomedical Sciences, Faculty of Medicine and Pharmacy, University of Oradea, 410087 Oradea, Romania; 2 Department of Pharmacy, Faculty of Medicine and Pharmacy, University of Oradea, 410028 Oradea, Romania; 3 Department of Preclinical Disciplines, Faculty of Medicine and Pharmacy, University of Oradea, 410087 Oradea, Romania; 4 Department of Medical Disciplines, Faculty of Medicine and Pharmacy, University of Oradea, 410087 Oradea, Romania; 5 School of Health Sciences &Technology, University of Petroleum and Energy Studies, Bidholi, Dehradun 248007, India

**Keywords:** sacubitril/valsartan, mortality, morbidity, heart failure, ejection fraction

## Abstract

Sacubitril/valsartan (S/V) is a pharmaceutical strategy that increases natriuretic peptide levels by inhibiting neprilysin and regulating the renin-angiotensin-aldosterone pathway, blocking AT1 receptors. The data for this innovative medication are mainly based on the PARADIGM-HF study, which included heart failure with reduced ejection fraction (HFrEF)-diagnosed patients and indicated a major improvement in morbidity and mortality when S/V is administrated compared to enalapril. A large part of the observed favorable results is related to significant reverse cardiac remodeling confirmed in two prospective trials, PROVE-HF and EVALUATE-HF. Furthermore, according to a subgroup analysis from the PARAGON-HF research, S/V shows benefits in HFrEF and in many subjects having preserved ejection fraction (HFpEF), which indicated a decrease in HF hospitalizations among those with a left ventricular ejection fraction (LVEF) < 57%. This review examines the proven benefits of S/V and highlights continuing research in treating individuals with varied HF characteristics. The article analyses published data regarding both the safeness and efficacy of S/V in patients with HF, including decreases in mortality and hospitalization, increased quality of life, and reversible heart remodeling. These benefits led to the HF guidelines recommendations updating and inclusion of S/V combinations a key component of HFrEF treatment.

## 1. Introduction

Heart failure (HF) is a prevalent disease these days, having a variety of etiologies. This syndrome involves the ventricle’s structure and function and affects patients’ quality of life, which means that work capacity, effort tolerance, sleep and psychosocial profile are altered. Even though HF is debilitating, and deadly, continuous research has developed more effective therapies. HF is the final stage of most types of heart disease. As a result, established risk factors play a crucial role in developing HF. High blood pressure, metabolic syndrome, low physical activity, dyslipidemia, and smoking [1,2] have all been tied to incident HF, either through coronary disease [3] or through conditions associated with HF, such as type 2 diabetes mellitus (T2DM) [4], chronic kidney disease [5] or overweight [6], which are widely known to be implicated in the genesis of HF via multiple pathways. HF describes symptoms and signs caused by cardiac abnormalities. HF most used terminology is the left ventricular ejection fraction (LVEF). HF with preserved ejection fraction (HFpEF) is described as HF with normal LVEF (≥50%) and HF with reduced LVEF (≤40%) as HF with reduced ejection fraction (HFrEF). Subjects that are HF-diagnosed, also having an LVEF of 40 to 49%, are known to have mildly reduced ejection fraction (HFmrEF) [7,8,9].

Decompensated heart failure (DHF) can be defined as an exacerbation of a chronic HF or an acute condition [10]. DHF usually appears in patients pre-diagnosed with HF and is characterized by signs and symptoms that are not tolerable and imply rapid therapeutic intervention [11]. However, DHF can arise de novo when it is triggered, among other causes, by complications of acute myocardial infarction (e.g., rupture of the chordae tendinae, acute mitral regurgitation, etc.), pulmonary embolism, and arrhythmias. This type of DHF is also known as acute heart failure (AHF).

The persistent burden of HF has lately been highlighted by data on cardiovascular mortality in the United States [12,13]. HF is significantly more common in older age groups, with a prevalence of 4.3% among 65–70 year-olds, and is expected to rise rapidly until 2030 when the incidence of HF might reach 8.5%. HF is usually included in elderly cardiovascular syndromes, which have a built-in burden of multiple chronic conditions and frailty, considerably exacerbating the disease’s personal and social costs. In addition, people of color with HF, especially women, have a disproportionately high impairment rate [14].

The New York Heart Association categorizes the relationship between dyspnea symptoms and physical activity: class I: no symptoms; class II: minor symptoms when engaged in regular physical activity; class III: patients still have no symptoms at rest but occur at a lower-than-normal activity; and class IV: extreme breathlessness even when patients are resting.

Drug therapy is gradually introduced according to the symptoms and stages of HF. Stage A (high risk, no symptoms) focuses on treating risk factors and comorbidities. Stages B (structural heart disease, symptoms missing) and C (structural heart disease, positive symptoms) require drug therapy. If bundle branch block is present, it should be considered cardiac resynchronization; if acute myocardial infarction is a problem, revascularization (PCI and CABG) must be performed. Finally, refractory symptoms require intervention in stage D: VAD (ventricular assisted device) and transplantation. Since neurohormonal involvement in HF has been recognized, there has been increased attention to the renin-angiotensin-aldosterone pathway (RAAS) and sympathetic activation. Thus, by suppressing the two, a decrease in mortality and rehospitalizations was demonstrated; moreover, some beta-blockers (BB) (carvedilol, nebivolol, prolonged-release metoprolol, bisoprolol) have also been shown to improve left ventricular function. Drug classes such as mineralocorticoid receptor antagonists (MRAs), angiotensin-converting enzyme inhibitors (ACEI)/angiotensin receptor blockers (ARBs) showed improved prognosis when combined [15,16,17,18].

Since the 2000s, a new range of drugs has been introduced in HF therapy [19,20,21,22], some of them having a spectacular evolution in terms of formulation [23]. A relatively new drug class that has made its presence felt in cardiology is co-transporter 2 (SGLT2) inhibitors, which has been proven as being effective in treating HFrEF, even if patients do not have DM; thus, doctors are encouraged to add this class of drugs (if they are not contraindicated or intolerated) to the treatment plan, among a beta-blocker ACEI, MRA, and an Angiotensin Receptor-Neprilysin Inhibitor (ARNI), to diminish the cardiovascular death risk or exacerbating HF. ARNI is known as combination of sacubitril and valsartan. 

Sacubitril and valsartan (S/V) formed this new drug class—ARNI, because ACEI and sacubitril combined produced significant angioedema. *Neprilysin* is an endopeptidase that degrades natriuretic peptides (NPs) and other endogenous vasoactive peptides. Sacubitril inhibits neprilysin, which raises the quantities of these peptides, and counteracts the opposite effect of neurohormonal overactivation [24]. The clinical efficacy of ARNI in HFrEF was proven in the prospective comparison of ARNI with ACEI to determine impact on global mortality and morbidity in HF (PARADIGM-HF) trial published in 2014 [25]. Experimental investigations have shown that inhibiting the RAAS and neprilysin simultaneously can reduce neurohormonal activation [26]. ACEI alone was inferior to ARNI in decreasing the hospitalization and the risk of death, in patients diagnosed with HFrEF in a double-blind large RCT [27].

MiRNAs are small molecules that can be identified at the blood level and are potential biomarkers to monitor in cardiovascular pathology, more precisely in heart failure [28]. These molecules are involved in cardiac adaptation processes. Some mechanisms involved in damaging the heart are fibrosis, hypertrophy, and apoptosis. These alterations at the cardiac level correspond to changes at the molecular level, so it is possible that in the future these genes will also be used as therapeutic targets. Published data [29] showed that ARNI increases the level of miRNA-18 and miRNA-145, which offers some protection against myocardial remodeling and oxidative stress at the cardiomyocyte level. Increased levels of miRNA-181 are associated with myocardial hypertrophy and fibrosis. ARNI has been shown to reduce the level of miRNA-181 [29].

In patients who have a poor therapeutic effect of cardiac resynchronization therapy with defibrillator (CRTd) and a worse prognosis, ARNI leads to significant improvement in clinical symptoms, cardiac pump, and reduction in NYHA class [29]. These effects ARNI-induced in CRTd patients lead to reduction in hospitalizations [29]. Notably, these effects are due to the regression of reverse cardiac remodeling via the modulation of microRNAs expression [29]. Indeed, the microRNAs are implied in the control of cardiac adaptive processes in CRTd patients [28], ARNI already being a common practice for HFrEF patients [25,30,31,32].

This study would like to present the modern therapeutic options, adapted according to the pathophysiological mechanism of the diseases, in HFrEF and HFpEF, and to emphasize the role of sacubitril/valsartan in heart failure therapy based on the data provided by the large trials. The review also aims to raise awareness to the medical public, starting from the general practitioner to the cardiologist, about the benefits of inclusion of S/V in the complex management of HF that led to its inclusion in the cardiology guidelines for heart failure.

## 2. The Pathophysiology of Heart Failure

Cardiac dysfunction, both structural and functional, causes decreased cardiac output and increased intracardiac pressures, which dictate the signs and symptoms of HF [7]. Cardiac injury, including myocyte cell loss, myocardial deformity, fibrosis, LV gradual dilation, and changes in ventricular shape, leads to cardiac remodeling, an imbalance in the demand/supply of oxygen from the heart, and altered contractility. In addition, arrhythmias also cause loss of heart pump function and systolic dysfunction [33]. Moreover, vasoconstrictor, pro-thrombotic and pro-inflammatory factors also contribute to cardiac injury, by altering diastole (both atrial and ventricular), so relaxation and filling of the cavities are no longer possible [34].

**Figure 1:**
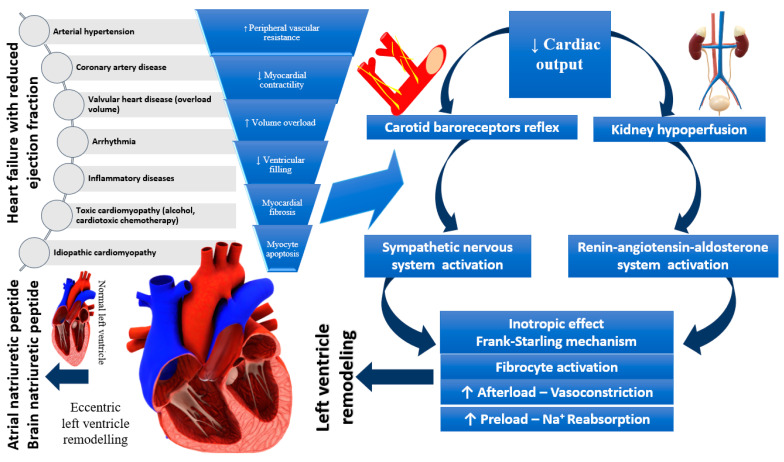
Pathophysiology of heart failure with reduced ejection fraction.

Considering only the ejection fraction, HF was divided into two major categories: HFpEF and HFrEF, which helped to make the diagnosis more accessible to establish the therapeutic course as quickly as possible, having an essential predictive value. Besides the two categories which consider EF < 40% and > 50%, a third category should be mentioned (that covers the gray area of 40–50%), namely HFmrEF. When more than one variable is considered, research has shown that myocardial dysfunction can be global, both systolic and diastolic, and that fibrosis, cardiomyocyte cell loss, oxidative stress, and coronary heart disease contribute at the onset of HF too. Moreover, the negative involvement of the RAAS in varying degrees in both types of HF has been shown, but the NPs counteracts the effects of RAAS by vasodilation, decreased wall thickness and inflammation of the heart, but also by its action on the nervous system [35]. Figure 1 demonstrates the relationship between the mechanisms described and HFrEF occurrence.

**Figure 2:**
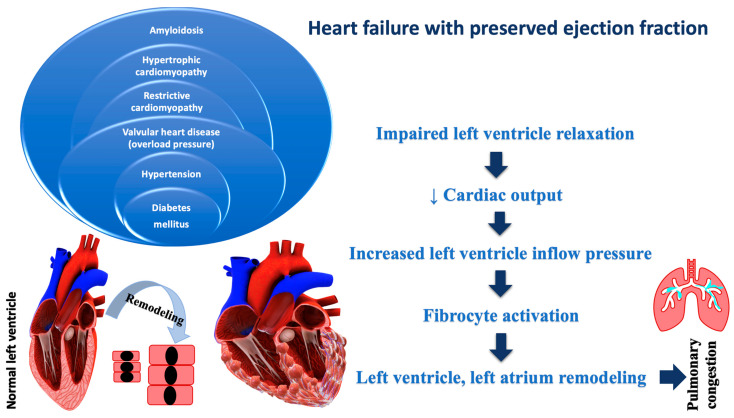
Pathophysiology of heart failure with preserved ejection fraction.

Evidence-based therapy improves symptoms and prognosis in HFrEF, and less in HFpEF. These variations underscore the importance of understanding the pathophysiological differences between HFrEF and HFpEF, which may influence therapeutic targets. The rising incidence and high mortality rates are standard features of both. It has been noticed that differences in giant spring titin, fibrosis, endothelial malfunction, and inflammation, as well as cardiomyocyte hypertrophy and apoptosis, vary in HF pathology. Cardiomyocyte hypertrophy, intercellular fibrosis, abnormal cardiomyocyte relaxation, and inflammation are all characteristics of HFpEF, resulting in the LV’s inability to relax adequately. Most of the time, HFpEF is associated with other chronic diseases, which can lead to its aggravation, and implicitly to an increase in the hospitalization rate. Non-cardiomyocytes are made up of about 60% endothelial cells, and endothelial dysfunction, which can be recognized early in cardiovascular disease, being less common in HFrEF vs. HFpEF. Several adaptive mechanisms can cause endothelial dysfunction in response to low cardiac output, such as vasoconstriction, nitric oxide imbalance, enhanced oxidative stress, neurohormonal activation and energy bioavailability [36]. Figure 2 illustrates the pathophysiology of HFpEF.

## 3. Diagnosis of Heart Failure and Types of Heart Failure

The HF presence is suggested by various signs and symptoms such as shortness of breath, cough, disrupted sleep, exercise intolerance, edema, and fatigue; in addition, displacement apex shock and increased jugular venous pressure may also be present. However, these variables are not always sufficient for the definite diagnosis of HF, because they can appear in other disorders as well (i.e., kidney failure, chronic obstructive pulmonary disease (COPD), and obesity) [7].

Thus, in addition to clinical evaluation and routine laboratory tests, specific laboratory tests are sometimes required—brain natriuretic peptides (BNP) and N-terminal proBNP (70% sensitivity, 99% specificity, respectively, 99% sensitivity, 85% specificity) [37]. The two cardiac biomarkers, BNP and proBNP, are secreted mainly by the ventricles but also by the atria. In the treatment of HF, BNP and NT-proBNP are known as having clinical importance in prognostic/diagnostic indicators. For example, BNP levels < 100 pg/mL had 90% predictive negative value during the diagnosis of HF in patients with acute dyspnea. In comparison, values > 500 pg/mL have >80% predictive positive value [38]. LVEF is a widely used phenotypic criterion for HF diagnosis. Both HFrEF (EF ≤ 40%) and HFpEF (EF ≥50%) are primary HF subtypes with distinct pathophysiology, etiology, and therapy outcomes. In addition, the proportions of the two phenotypes with specific risk factors differ. HFpEF, for example, is defined by female gender and advanced age [39,40,41,42,43].

Comorbidities, numerous risk factors, and pre-existing illnesses contribute to HF, harming heart’s function and structure. The first choice of HF medications aimed to improve quality of life by reducing morbidity and death in HFrEF while reducing symptoms and slowing disease progression. However, to date, no HF therapy has been reported to reverse (constantly and permanently) the evolution of structural and functional degeneration of the heart [7,44]. Despite breakthroughs in treatment, the prognosis in HF remains poor. Patients with HFpEF exhibit symptoms and signs of HF, and proof of cardiac dysfunction as a source of symptoms [2]. HFpEF has the same clinical symptoms as typical HF, including HFrEF [45]. HFpEF refers to those having HF signs/symptoms of HF, or cardiac abnormalities (LV diastolic dysfunction/increased left ventricular filling pressures, and/or elevated NPs, as well as an LVEF above 50%) [46,47].

## 4. Pharmacologic Therapy for Heart Failure

### 4.1. Treatment in HFrEF

The decrease in cardiac output causes an inadequate circulating volume that will be ameliorated, at the onset of HF, by the compensatory mechanisms through neurohormonal involvement: sympathetic activation, RAAS, and release of antidiuretic (ADH). Chronic activation of these mechanisms will cause vasoconstriction and fluid retention, depletion of catecholamines, and a weak response to the action of circulating catecholamines, contributing to myocardial hypertrophy and cardiac remodeling, which are present in HF [48,49,50]. ACEI impacts significantly the neurohormonal state of HF subjects by interfering with the RAAS by limiting angiotensin I (ATI) to convert to angiotensin II (ATII), causing vascular relaxation, decreased vasoconstriction and vascular resistance [51,52,53,54].

Low levels of ATII promote the elimination of Na from the body, decreased vasoconstriction and blood pressure (BP). These effects are due to decreased sympathetic activity, ADH production and aldosterone. Low preload and afterload results from low venous and arterial pressure lead to improved ventricular filling and better blood ejection. ACEI can help prevent ventricular remodeling by limiting cardiac hypertrophy and myocardial fibrosis and reducing cardiomyocyte death by acting at the cellular level. ACEI have been demonstrated to have positive benefits in chronic HF [55,56,57,58].

In patients with HFrEF, ACEI enhance symptoms, life quality, and physical function. When compared to placebo, ACEI reduced death by 23% and HFrEF-related mortality or hospitalization by 35%, according to an analysis of 32 randomized clinical studies in people with HFrEF. ACEI improve survival and lower the risk of HF and coronary events in patients with a reduced LVEF, but no HF [59,60,61].

Angiotensin receptor blockers (ARBs) suppress the RAAS by blocking angiotensin II from binding to its receptor, preventing constriction of the blood vessels and aldosterone release; ARBs do not inhibit kininase, which lowers cough compared to ACE inhibitors. Therefore, ARBs should be used to minimize morbidity and fatality in those patients which cannot be administered ACEI (considering their side effects) [62], or in patients in whom ARNI is not feasible, according to the 2022 ACCF/AHA/HFSA guidelines [63]. Furthermore, due to the danger of cross-reaction, ARBs should be administrated with caution in the case of the subjects having in their medical history of angioedema induced by ACEI [64].

According to the guidelines, in patients having HFrEF NYHA class II/III, ARB medication should be replaced with an ARNI [65]. The Candesartan in Heart Failure (CHARM Alternative) study compared candesartan to placebo and found that candesartan improved cardiovascular outcomes compared to placebo, including cardiovascular death or hospital readmission. In comparison to 40% of placebo patients, only 33% of candesartan patients died of cardiovascular cause or were hospitalized for HF [66].

RAAS inhibitors have been shown to have cardioprotective effects. In the study published by Marfella et al. in 2022, the effect of RAAS blockers was analyzed in heart transplant patients with/without T2DM. When the research started, no significant differences were observed in terms of myocardial fibrosis, but at one year of follow-up, there were differences between patients who did not have T2DM and those who were diagnosed with T2DM, and more than that, differences were observed in patients who had a more rigorous glycemic control. It should be clarified that all patients followed a similar therapy with ACEI or ARB. The study evaluated the involvement of Ang 1–7 and Ang 1–9 molecules responsible for the antifibrotic effects at the cardiac myocyte level and observed that their levels are higher in patients without T2DM and in patients with better glycemic control [67].

Beta-blockers bind to beta-adrenoceptors and prevent adrenaline and noradrenaline from binding to these receptors, reducing the SNS’s functions. Fundamental studies have shown that giving a BB to symptomatic patients with decreased LVEF reduces mortality and morbidity. In the SENIORS, MERIT-HF, COPERNICUS, and Cardiac Insufficiency Bisoprolol Study (CIBIS)-II trials, this was proved with nebivolol, carvedilol, bisoprolol, and controlled-release metoprolol [68,69,70,71].

Because of detrimental inotropic effects, it is not indicated that BB be started during an HF exacerbation; instead, this class is recommended when the patient is volume-stable [22,72]. Only certain BBs reduce mortality and rehospitalizations because BBs do not show a class effect. The effects of bisoprolol and metoprolol CR/XL were compared to placebo and resulted that there has been a reduction of all generating causes of mortality, hospitalizations, and even NYHA functional status [71,73].

Mineralocorticoid receptor antagonists work by inhibiting the mineralocorticoid receptor, which counteracts the effects of aldosterone since MRAs provide diuretic properties and can contribute to a fluid balance. The main beneficial effects are observed in decreasing mortality, respectively, the number and duration of hospitalizations due to HF (explainable by both decreasing the risk of hypokalemia, prevention of myocardial/renal fibrosis caused by excess aldosterone, etc.) [74,75,76]. Spironolactone side effects (e.g., gynecomastia) are caused by the affinity of spironolactone for glucocorticoid, progesterone, and androgen receptors. Eplerenone does not determine gynecomastia, so it is a better choice, but a higher dose is needed, because of the lower affinity [77,78,79].

Sodium-glucose cotransporter-2 inhibitors (SGLT2i) consider a newer class of drugs, with antidiabetic effect, that improve natriuresis and osmotic diuresis, while increasing the excretion of glucose in the urine to reduce blood glucose levels.

The Dapagliflozin and Prevention of Adverse-outcomes in Heart Failure Trial (DAPA-HF) randomized trial with dapagliflozin was designed in the HFrEF group, even if patients did not have DM [80].

When compared to placebo, the study main purpose was to see how adding 10 mg of dapagliflozin to the optimal treatment for HFrEF affects the primary endpoint’s occurrence, aggravation of HF (decompensated HF) and cardiovascular mortality. The dapagliflozin-treated group exceeded the placebo-treated group in every parameter tested throughout the course of an average 18-month follow-up. In addition, concerning the first exacerbating HF event and death from cardiac or other causes, the group using dapagliflozin (as treatment) had a reduced risk. Empagliflozin improves physical performance and life quality, while lowering the risk of HF hospitalization in the Empagliflozin Outcome Trial in Patients with Chronic Heart Failure with Reduced Ejection Fraction (EMPEROR-Reduced study) [81].

In HFrEF patients, the two approaches joined (inhibition of NP breakdown and blockage of the RAA) proving to be the most effective together [82]. In real-life and contemporary research, ARNI has established itself as a first-line drug in the treatment of HFrEF. Its favorable effect on cardiac remodeling has also been confirmed. The major objective of the Rationale and Methods of a Prospective Study of Biomarkers, Symptom Improvement, and Ventricular Remodeling During Sacubitril/Valsartan Therapy for Heart Failure (PROVE-HF) trial was to find a relationship between changes of cardiac remodeling and NT-proBNP concentration values [83]. In the PRIME trial (Pharmacological Reduction of Functional, Ischemic Mitral Regurgitation), another study found that valsartan alone was inferior to ARNI in standard therapy, in reverse remodeling and downsizing functional mitral regurgitation (FMR) in a group of participants diagnosed with HF with an EF ≈ 25–50%, symptomatic (NYHA II-III) and significant FMR lasting more than 6 months. After 12 months, in the group treated with ARNI, a reduction in EROA (effective regurgitant orifice area) was noticed to be more significant than in the group treated with valsartan; this was the main indicator of FMR improvement. Reducing the effective regurgitant orifice area determined a reduction in the end systolic/diastolic volume of the LV, in both groups, S/V, respectively, valsartan. The S/V group outperformed the valsartan group in regurgitant volume (mean difference, 7.3 mL). Additionally, the S/V group had a substantially higher decline in diastolic LV function−E/e′. These data support S/V role in cardiac reverse remodeling [84].

New information concerning the mechanisms of this drug class in HFrEF was revealed after researchers analyzed the effect on inflammation, functional ability, and peripheral vascular activity. A group of patients with HFrEF (LVEF~28%) were administered S/V and investigated prospectively. Patients were evaluated at the beginning of treatment, then monthly for up to three months, and a decrease in pro-inflammatory markers, improvement in functional capacity and peripheral vascular activity were observed [85].

### 4.2. Treatment in HFpEF

The development and progression of HFpEF are linked to abnormal activation of the RAAS. In addition, the RAAS reduced LV diastolic performance (increasing myocardial/arterial stiffness) and caused LV hypertrophy [86,87]. As a result, multiple randomized clinical trials have assessed the RAAS blockade’s prognostic value (CHARM-preserved, PEPCHF, I-PRESERVED). Unfortunately, the results were not as optimistic as expected: candesartan and perindopril reduced hospitalization rates due to HF and symptoms, but irbesartan did not reduce hospitalizations and patients’ quality of life did not improve in the long term [88,89,90,91]. Due to their effectiveness in HFrEF, attempts have been made to initiate BB in treating HFpEF. Given that there is also a ventricular filling defect in HFpEF due to adrenergic stimulation, BB may be helpful in decreasing the adrenergic response and HR and improving exercise tolerance [92,93].

Aldosterone has implications for the development of myocardial fibrosis that will cause adverse effects on the heart muscle. MRAs act on aldosterone receptors and are useful in treating HF regardless of the left ventricular ejection fraction. The ALDO-HF study proved the usefulness of this class in HFpEF from a cardiac point of view but not showing improvements in symptoms or in the life’s quality [94]. The TOPCAT-HF trial studied the effects of spironolactone compared to placebo, but the results were not encouraging. In the group treated with spironolactone, the unwanted effects did not take long to appear, so hyperkalemia and renal failure were present to a higher degree. However, this trial opens the way for other studies regarding HFpEF therapy, depending on the etiology [95].

ARNI combines RAAS blockade and endogenous natriuretic peptide pathway upregulation. In HFrEF, S/V is an emerging and game-changing disease-modifying drug. Inhibition of neprilysin increases endogenous natriuretic/vasoactive peptides, such as cGMP, which are reduced in HFpEF and linked to myocardial stiffness. In a phase II study of patients with HFpEF, S/V was linked to LA reverse remodeling, a more considerable reduction in NP levels, and symptoms improvement [96,97]. Patients with HFpEF may benefit from this discovery. To determine the efficacy of S/V in HFpEF, the Prospective Comparison of ARNI with ARB Global Outcomes in HF with Preserved Ejection Fraction (PARAGON-HF) trial was conducted. Subjects having LVEF > 45% and NYHA II-IV were included in the study. The study’s findings were not as statistically significant as expected, but it was found that S/V is more effective among women [98,99,100].

## 5. Sacubitril/Valsartan Therapy

### 5.1. Sacubitril/Valsartan in HFrEF—Evidence of Efficacy in Clinical Trials

In the last three decades, HF treatment has experienced an unparalleled evolution, based on evidence, with an increase in the quality of living and the survival of patients suffering from HF. International guidelines support this progress, especially the ESC Guide. Compared to the guide published in 2016, the 2021 guide focuses on patient involvement in managing the treatment of their disease, of course with a multidisciplinary team. Along with conventional therapy (ACEI, MRAs, beta-blockers), ARNI and SGLT2i are added to the therapeutic regimen of subjects with HFrEF. These medication classes have both independent and additive therapeutic effects [46,101].

In the PARADIGM-HF, patients with HFrEF and NYHA class II, III, or IV HF have been randomly selected to be administered either S/V or enalapril, in addition to standard therapy. The study aimed to identify the variations in mortality rates from CV causes. The study ended earlier in agreement with predetermined rules because the threshold for an overwhelming advantage with S/V had been crossed. A total of 17% of patients treated with S/V and 19.8% with enalapril died, but S/V reduced the rehospitalization rate due to HF by 21% and improved symptoms. Hypotension and angioedema were more pronounced in the ARNI group, but fewer patients developed coughs, electrolyte imbalances, and kidney damage [25]. A blinded independent committee conducted and adjudicated a meticulous and highly extensive investigation of the mode of death subsequent to the first release of PARADIGM-HF. The mortality causes were divided into non-CV or CV deaths. In CV deaths, it was observed that S/V reduced the death rate by 20% compared to the control group treated with enalapril [102].

In the PIONEER-HF trial, subjects suffering from HFrEF hospitalized due to IC decompensation were randomized into two groups: one was treated with ARNI and another with enalapril. Shortly afterward, a more considerable reduction in NT-proBNP levels was observed in the group treated with ARNI. The decrease in NT proBNP, which indicates neurohormonal activation and hemodynamic stress, was linked to a decrease in high-sensitivity cardiac troponin, indicating myocardial damage. Compared to enalapril, S/V subjects were unlikely to be re-hospitalized for HF at eight weeks. This trial was the first to show that starting S/V in the hospital was tolerable and safe [103].

The goal of the TITRATION trial was to offer information on how to start and up titrate S/V in people with chronic HFrEF. TITRATION included patients who had never been treated before or had varying levels of ACEI/ARB pre-treatment [33,42]. Patients were divided into two groups: one for concentrated titration, where the dose was increased in three weeks, and one for conservative titration, where the dose was increased in six weeks. By measuring the patient’s tolerance to treatment, therapeutic success was obtained in both groups analyzed. Patients who have not received any ACEI/ARBs previously can tolerate S/V if introduced gradually, according to the TITRATION trial. It was observed that if the higher dose is not tolerated in the beginning, a down titration can be beneficial; this technique can allow the physicians to reach the target dose in time. The TITRATION trial reveals that S/V can be titrated in most of the subjects in three weeks, excluding the patients naïve to ACEI/ARBs therapy [104].

PRIME study aimed to show that S/V has a favorable effect on remodeling in HFrEF patients. In the PRIME study patients suffer from chronic functional mitral regurgitation due to left ventricular malfunction and reduced EF. These subjects received guideline-directed medical therapy in a double-blind experiment. Valsartan or S/V were given to the patients. The S/V group developed a decrease in EROA, thus proving that the drug has a better effect on heart remodeling than the valsartan group; additionally, an improvement in the regurgitant volume was noticed. Some other noticeable results show that the S/V group had a higher decrease in LV end-diastolic volume index, but on partial mitral leaflet closure area, other measures of the LV, or changes in blood pressure, it was not a remarkable difference. PRIME is considered a modest trial being the first to demonstrate the reverse remodeling impact of S/V in people with HFrEF, associated with suffering from FMR [84].

In the PARADIGM-HF study, a lower NT-proBNP level was linked to a better result in patients using S/V. The PROVE-HF wanted to investigate this further because NT-proBNP lowering during guideline-directed healing therapy has already been associated with cardiac remodeling reversal. PROVE-HF was open-label research in which 794 individuals suffering from chronic HFrEF were distributed to S/V and had their echocardiogram carried out before starting treatment, six months later, and 1 year later. At each study visit, the concentration of NT-proBNP was assessed. Following the completion of all research procedures, at a core laboratory, echocardiograms were blindly analyzed temporally and clinically. After starting S/V, the researchers found a significant 37 percent reduction in NT-proBNP and reverse cardiac remodeling intimately tied to it. At the starting point, the LVEF was ~28%, but after 12 months of treatment, it increased by 9.4%, with a significant improvement in other patients. Additionally, more variables were modified: diastolic function, measured by E/e′ proportion, improved, and the mass index of LV and volumes of LV and LA reduced. These results are maintained in patients who were newly diagnosed with HF or those who had not previously been treated with ACE inhibitors or ARBs or patients who did not achieve the target dose of S/V. The PROVE-HF research adds to the growing body of evidence supporting reversible cardiac remodeling and the drop of NT-proBNP levels when ARNI is administered [105].

Whether aortic impedance can contribute (from the pathophysiologic point of view) in patients diagnosed with HFrEF and treated with ARNI was one of the questions answered in the EVALUATE-HF trial. Patients were divided into two groups: one that received S/V and the other one, enalapril. It resulted that the S/V group showed a decreased aortic impedance, while the valsartan’s group was increased; still, the dissimilarity was not eloquent. Compared to the enalapril group, the S/V group exhibited considerably lessened NT-proBNP levels and a significant reduction in numerous echocardiographic parameters (LVED, SVI, LAVI mitral E/e′ index). An exploratory secondary goal was to show a substantial increase in the general summary score for the 12-module Kansas City Cardiomyopathy Questionnaire (KCCQ). Despite three months of S/V medication, these findings demonstrate a clear remodeling benefit compared to usual care. The EVALUATE-HF research found that, while no considerable improvement was observed when administering S/V in aortic impedance, in contrast with enalapril, it did show more substantial cardiac reverse remodeling and improved quality of life [106].

A summary of the results obtained from the above presented clinical trials is presented in the Table 1.

Associations between S/V post-discharge adherence (PDC) and clinical outcomes after readmissions for HFrEF revealed that 32.9% patients who received ARNI therapy were compliant when discharged (PDC ≥ 80%), but 67.1% were not compliant to the treatment (PDC < 80%). Between groups, baseline attributes were evenly distributed. Subjects with PDC ≥ 80% presented considerably decreased adjusted risk of all-cause readmission to hospital and mortality after 90 days and one year compared to patients with PDC < 80%. At one year, patients saw a significant reduction in rehospitalization for every five-percentage point rise in PDC. Finally, the trial proved that compliance to the therapy with S/V, 90 days after discharge was related to diminished rehospitalization rates and death in patients hospitalized for HFrEF and discharged on S/V. In HFrEF, more work is needed to increase adherence to S/V and other guideline-directed medical therapy [109].

### 5.2. Sacubitril/Valsartan in the Treatment of HFpEF

The researchers assigned HF patients with LVEF > 45%, NYHA II-IV, high NP values, and structural heart disease to receive target doses of S/V and valsartan (97/203 mg × 2/day, respectively 160 mg × 2/day). Renal function, safety, functional class, KCCQ score, CV death, and HF hospitalizations were monitored. The results favor S/V except for hospitalizations and CV deaths, where there was no statistically significant difference [98].

A randomized, parallel-group, double-blind clinical experiment included subjects with HF, LVEF > 40%, high NT-proBNP concentrations, structural heart disease, and bad quality of life registered from 396 facilities in 32 countries. The aim of the research was to compare the results of S/V on NT-proBNP levels, 6-min walk test (6MWT) and life quality in subjects suffering from chronic HF and LVEF greater than 40% to background medication-based individualized comparators. The trial was completed by 87.1% of patients. At the baseline, the levels of NT-proBNP were comparative in both groups (786 pg/mL—S/V group, respectively, 760 pg/mL—comparative group). After 12 weeks, the group that received S/V had a considerable reduction in NT-proBNP levels compared to the other group. A big difference was not noticed regarding the 6MWT after 24 weeks (at the baseline 9.7 m and at the end 12.2 m) nor in NYHA class and KCCQ score (12.3 vs. 11.8, respectively 23.6% vs. 24.0% of patients). S/V treatment determined a more notable decrease in plasma NT-proBNP peptide concentrations compared to standard RAAS inhibitor treatment or placebo. However, it did not significantly enhance the 6MWT in subjects suffering from HF and a LVEF > 40% [110].

A systematic review on four randomized controlled studies of S/V for HFpEF patients showed a reduced rate of HF hospitalization in HFpEF patients when compared to the control group. S/V did not exhibit any clear benefits in cardiovascular mortality, mortality of all causes, or improvement in the NYHA class. Even though S/V was connected to an increased risk of symptomatic hypotension, there were no signs of deteriorating renal function or hyperkalemia. Except for the hospitalization rate, in which the S/V treatment group favored HFpEF patients, the study found no differences between the two groups compared to valsartan or personalized medical therapy [111].

Previous research has validated S/V’s efficacy in treating HFrEF. However, the role of S/V in HFpEF is still an area of research. S/V is a combination therapy that includes sacubitril and valsartan and functions as neprilysin inhibitor (ARNI) and a first-generation angiotensin receptor blocker. Using animal models, the effect of S/V was evaluated in a high sodium diet that causes HFpEF and vascular damage, in addition to the primary mechanism. Thus, a considerable amount of salt (68 mg/kg) was administered intragastrically before S/V treatment. According to the findings of functional tests, it has been observed that a high sodium diet leads to lesions in the heart and vascular system. These effects were reversed by S/V administration; in addition, S/V had an antifibrotic impact by decreasing the amount of type 1 and type 3 collagen and decreasing the MMP3/SMAD3 ratio. The most plausible explanation for the favorable effects on HFpEF is the action of S/V on the TGF1/SMAD3 signaling pathway. Following this experiment, S/V proved its therapeutic potential in HFpEF, reversing the effects of HFpEF induced by a high sodium diet. However, this study had some drawbacks: to corroborate the findings, the study did not identify the agonists or antagonists of the TGF-/Smad signaling pathway; instead, it merely examined the biochemical effects of a high-salt diet and S/V. HFpEF is still controversial in high salt-induced rat models even after thorough reports by the team of Y Sakata/M Hori. For the thorough verification of these data, in vitro experiments are also necessary [112]. S/V has been given a pioneering expanded indication by the FDA, making it the first medicine in the US to be approved for chronic HF that is not defined by ejection fraction.

### 5.3. Sacubitril/Valsartan in Advanced Heart Failure

According to estimates, 1% to 10% of people with heart failure have advanced heart failure [113]. However, the prevalence is growing due to the rising number of people with heart failure and improved survival and treatment [114]. Advanced heart failure is characterized by low ejection fraction (LVEF ≤ 30%), right ventricle failure, congenital or valve abnormalities that are not operable, high values of the cardiac biomarkers and low to very low quality of life of the patients (e.g., NYHA III/IV, inability to fulfill 300 m without symptoms). Therefore, this stage of HF can be classified as stage D according to ACC/AHA classification. In addition, advanced HF involves repeated interventions by the medical staff and more aggressive therapy to manage the severe overlapping symptoms [46,115]. Researchers analyzed the medical records of all severe HF patients who were assessed at their center for heart transplant therapy. They looked at individuals who had started ARNI medication and had their hemodynamics checked before and after six months. At six months after initiating ARNI medication, the first noticed result was pulmonary pressures and filling pressures variety. Systolic pulmonary artery pressure (32 mm Hg vs. 25 mm Hg) in addition to average pulmonary artery pressure (20 mm Hg vs. 17 mm Hg) were significantly lower six months after commencing ARNI. Because of improvement, 23% patients were removed from the heart transplant list, while four new patients were added to the list. After almost two years, three patients were treated with a left ventricular assist device, while six patients had their hearts transplanted. S/V was found to be safe and effective in lowering filling pressures and pulmonary pressures in subjects suffering from advanced HF [116]. Advanced HF in patients with reduced EF NYHA IV was not an inclusion criterion in the large clinical trials PARADIGM-HF and PIONEER-HF, so in these patients, the effectiveness of S/V in their treatment could not be confirmed. To assess the efficacy and impacts of S/V, the randomized clinical trial LIFE (LCZ696 In Hospitalized Advanced Heart Failure) reached an answer [117]. Therefore, the LIFE trial tried to objectify the efficacy and effects of S/V through the changes made to NT-proBNP levels from the beginning of the study to 24 weeks of therapy. The study included 335 patients with this pathology who were administered S/V or valsartan (target dose—200 mg × 2/day, respectively, 160 mg × 2/day). The trial showed that there are no significant differences between the two groups [118]. Moreover, in patients whose tolerability was not verified to one of the ACEI/ARB classes before initiating S/V, it was proven that 50% of patients would have an intolerance to S/V. Therefore, this study concludes that the benefit of S/V in patients with HFrEF in an advanced stage must be weighed against the risk [117].

## 6. Ongoing Research with Sacubitril/Valsartan in the Treatment of Heart Failure

The first study (PARAGLIDE-HF) is a multicenter, randomized, and double-blinded clinical trial that is expected to enroll 450 participants. Following hospitalization for acute decompensated HF, this study will assess the effects of S/V compared to valsartan monotherapy on clinical outcomes, safety, tolerability and NT-proBNP levels in decompensated HFpEF patients that were stabilized, and the treatment with S/V will be instituted at the time or in 30 days after patient stabilization [119]. 

A total of 50 people will be enrolled in the second study. It will look at the effects of S/V on the autonomic cardiac nerve system in HF patients by measuring HR variability [120].

The third study will assess the effects of S/V vs. valsartan on cardiac oxygen demand and cardiac work efficiency in 60 participants with NYHA II-III HFrEF after six weeks of therapy [121].

The fourth trial will enroll 48 patients and will examine S/V vs. placebo in terms of neurohormonal activation and physical function in people presenting right ventricular dysfunction (moderate/severe) and those that have present NYHA II-III symptoms [122]

The researchers will enroll 100 patients in the fifth study, which will be a phase 3 randomized controlled trial to see if S/V can reverse heart hypertrophy and fibrosis [123].

The SHORT trial, the sixth study, is a randomized experiment on HF patients with LVEF < 35%. Its goal is to see if a quicker protocol translates to faster optimization and a higher degree of optimization than the current standard protocol [124].

A multicenter prospective randomized study will assess the effect of in-hospital initiation of S/V on the NT-proBNP concentrations in patients admitted due to AHF (PREMIER) and is estimated to include 400 participants [125].

## 7. Current Guidelines Recommendations

Recent ACC AHA 2022 HF recommendations indicate the use of ARNI as first line therapy to lessen morbidity and fatality in subjects suffering from HFrEF and those that present NYHA class II/III symptoms. Subjects with HFrEF, NYHA II-III that are under treatment with ACEI/ARBs should be switched to ARNI due to favorable morbidity and mortality results. ARNI is indicated as a de novo medication in subjects suffering from AHF and chronic symptomatic HFrEF, given the improved prognosis, decreased NTproBNP value, and cardiac remodeling [44].

**Figure 3:**
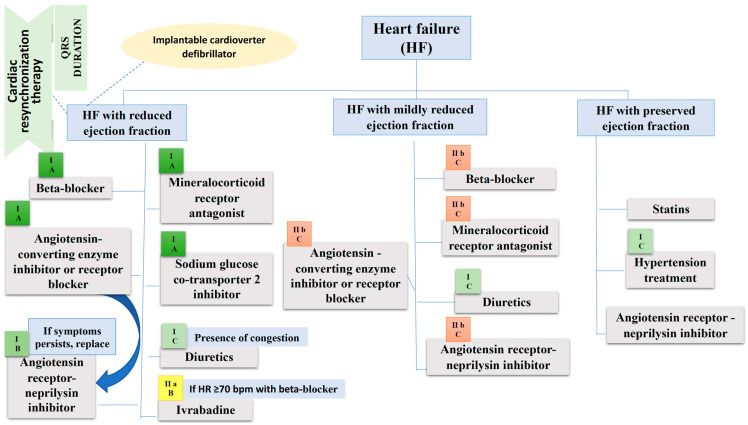
Management of heart failure according to the European Society of Cardiology guideline. Beside each therapeutic agent there is the class and level of recommendation mentioned in the European Society of Cardiology guideline. In HF with preserved ejection fraction the levels of recommendation for drugs included in the figure are not clearly specified because some trials were not published by the time the guideline has been released, so we cannot consider them class I treatment options. b.p.m., beats per minute; HR, Heart rate.

From the perspective of mortality and morbidity, none of therapies described in this review have been shown efficient in HFpEF; it is crucial to understand the mechanisms underlying this condition. However, the PARAGON-HF study revealed a lower readmissions rate in those patients with HF and LVEF < 57%, and a systematic review that included PARADIGM-HF and PARAGON-HF trials showed a decrease in CV mortality and HF hospitalization in patients that had an LVEF lower than the normal interval [46]. FDA granted S/V, an indication to treat patients with HFpEF. Management of HF according to latest guideline recommendations is illustrated in the Figure 3.

## 8. Conclusions

S/V is a breakthrough drug in HF therapy. Compelling benefits of ARNI therapy in HFrEF were demonstrated in PARADIGM HF with a reduction in CV mortality and hospital admission for HF, and a decrease in all-cause mortality. Sudden cardiac death was reduced by 22%, as were all-cause and HF readmissions and AHF. S/V reduced symptoms and physical constraints linked to HF, as assessed by the KCCQ, and this advantage expanded to almost all areas of the score when examined individually. There is an expanding body of evidence supporting S/V’s role in cardiac reverse remodeling process correlated with a significant reduction of NT-proBNP level in EVALUATE HF and PROVE HF. The PRIME study demonstrates for the first time the result of S/V treatment on reverse remodeling in HFrEF subjects with functional mitral regurgitation. S/V decreased the rate of cardiovascular mortality and readmissions in subjects suffering from HFpEF in PARAGON-HF, though the results are not statistically significant. In addition, advantages for life quality and renal function have been reported, but also from a clinical perspective. S/V treatment reduced the symptoms of HFpEF produced by a high-salt diet, most likely by decreasing fibrosis, highlighting S/V’s therapeutic promise for HFpEF. For patients admitted to hospital for HFrEF and discharged on S/V, increased adherence to therapy was connected to considerably lower readmission rates and death.

## Figures and Tables

**Table 1:** Clinical trials main information.

Trial Acronym [Ref], Setting	Study Design Patients’ no./Inclusion Criteria Period	Results
PARADIGM-HF [25], ambulatory preceded	S/V (target dose 97/103 mg × 2/day) vs. enalapril; a multicenter, prospective, randomized clinical trial 8442/NYHA II–IV (EF ≤ 40%) follow-up of 27 months	CVD mortality ↓20% HF hospitalization ↓21%
TITRATION [104], ambulatory	S/V clinical trial, multicenter, prospective, randomized/ 498/NYHA II–IV (EF ≤ 35%)/ 16-week study period	76% of patients achieved and maintained S/V without modifying the dose 77.8% of patients reached the appropriate S/V dosage, 84.3% with a prolonged titration Safety was equal
PRIME HF [84], ambulatory	S/V vs. valsartan in a multicenter, prospective, randomized clinical study 118/HFrEF (EF < 50%) 12-month study period	reduction in EROA significant changes in regurgitant volume and LVEDV no significant changes in LVESV and inadequate mitral leaflet closure area.
EVALUATE-HF [106], ambulatory	S/V vs. enalapril in a multicenter, prospective, randomized clinical trial 464/ HFrEF (EF ≤ 40%) S/V or enalapril were assigned at random. 12-week study period	difference in aortic characteristic impedance—not substantial changes in LAVI, LVEDV, LVESV, mitral E/e′ ratio EF increased by 1.9% in S/V group
PROVE-HF [105], ambulatory	S/V clinical trial, multicenter, prospective, open label 794/HFrEF (EF ≤ 40%) 12-month study period	reduction in NT-proBNP changes in LVEDV, LVESV, LAVI and E/e′ ration reversal cardiac remodeling low NT-proBNP, not attain target dose, new-onset HF, or not taking an ACEI/ARB at the time of enrolling—similar outcomes.
PIONEER-HF [107], in-hospital	S/V was compared to enalapril in a multicenter, prospective, randomized clinical trial 881/NYHA II–IV (EF ≤ 40%) 8-week study period	47% against a 25% drop in NT-proBNP S/V is safe in AHF or new-onset HF recurrent HF hospitalizations—reduced no significant difference between the two groups regarding renal function, hypotension, and hyperkalemia
TRANSITION [108], in-hospital	A multicenter, prospective, randomized clinical trial 1.002/HFrEF (EF ≤ 40%)	patients who reached the target dose of S/V was similar S/V safe and well-tolerated in patients with AHF and new-onset HF

AHF, acute heart failure; EF, ejection fraction; HF, heart failure; HFrEF, HF with reduced ejection fraction; LVEDV, left ventricle end-diastolic volume; LVESV, left ventricle end-systolic volume; NYHA, New York Heart Association; S/V, sacubitril/valsartan; LAVI, left atrial volume index; ↓ reducing.

## Data Availability

Not applicable.
